# Preoperative Bone Loss Predicts Decreased Survival Associated with Microvascular Invasion after Resection of Hepatocellular Carcinoma

**DOI:** 10.3390/cancers16112087

**Published:** 2024-05-30

**Authors:** Takashi Ishida, Atsushi Miki, Yasunaru Sakuma, Jun Watanabe, Kazuhiro Endo, Hideki Sasanuma, Takumi Teratani, Joji Kitayama, Naohiro Sata

**Affiliations:** Department of Surgery, Division of Gastroenterological, General and Transplant Surgery, Jichi Medical University, Shimotsuke 329-0498, Tochigi, Japan; taishida@jichi.ac.jp (T.I.); naruchan@jichi.ac.jp (Y.S.); m06105jw@jichi.ac.jp (J.W.); kendo@jichi.ac.jp (K.E.); h-ssnm@jichi.ac.jp (H.S.); teratani@jichi.ac.jp (T.T.); kitayama@jichi.ac.jp (J.K.); sata2018@jichi.ac.jp (N.S.)

**Keywords:** biomarker, osteopenia, Hounsfield unit, sarcopenia, myosteatosis

## Abstract

**Simple Summary:**

The aim of this study was to elucidate whether osteopenia predicts clinicopathological findings in patients with hepatocellular carcinoma. Bone mineral density was assessed using computed tomography scan images taken within 3 months before surgery. The cutoff value of osteopenia was calculated using a threshold value of 160 Hounsfield units. The overall survival of osteopenia patients was shorter than that of non-osteopenia patients, regardless of gender. Osteopenia was an independent risk factor for overall survival and recurrence-free survival. The pathological factor associated with osteopenia was microvascular portal vein invasion.

**Abstract:**

Background: Osteopenia is a well-known risk factor for survival in patients with hepatocellular carcinoma; however, it is unclear whether osteopenia can apply to both genders and how osteopenia is associated with cancer progression. The aim of this study was to elucidate whether osteopenia predicts reduced survival in regression models in both genders and whether osteopenia is associated with the pathological factors associated with reduced survival. Methods: This study included 188 consecutive patients who underwent hepatectomy. Bone mineral density was assessed using computed tomography (CT) scan images taken within 3 months before surgery. Non-contrast CT scan images at the level of the 11th thoracic vertebra were used. The cutoff value of osteopenia was calculated using a threshold value of 160 Hounsfield units. Overall survival (OS) curves and recurrence-free survival (RFS) were constructed using the Kaplan–Meier method, as was a log-rank test for survival. The hazard ratio and 95% confidence interval for overall survival were calculated using Cox’s proportional hazard model. Results: In the regression analysis, age predicted bone mineral density. The association in females was greater than that in males. The OS and RFS of osteopenia patients were shorter than those for non-osteopenia patients. According to univariate and multivariate analyses, osteopenia was an independent risk factor for OS and RFS. The sole pathological factor associated with osteopenia was microvascular portal vein invasion. Conclusion: Models suggest that osteopenia may predict decreased OS and RFS in patients undergoing resection of hepatocellular carcinoma due to the mechanisms mediated via microvascular portal vein invasion.

## 1. Introduction

Hepatocellular carcinoma is the second-most common malignancy and fifth-most common cause of cancer-related death worldwide [1]. International guidelines provide detailed treatment options for each stage of hepatocellular carcinoma depending on patient liver function and tumor burden, ranging from curative treatments such as hepatectomy, transplantation, ablation, and combination therapy to palliative treatments such as transcatheter arterial chemoembolization (TACE), systemic therapy, and supportive care [2]. Liver resection is a curative option for patients with hepatocellular carcinoma who meet the criteria, but most (70–80%) patients develop tumor recurrence or metastasis after hepatic resection [3]. The high rate of recurrence may be due to the high incidence of intrahepatic metastases and the multicentric nature of de novo HCC [4]. Patient prognosis in hepatocellular carcinoma is related to many factors, including tumor features and liver function [5], and due to its high recurrence rate, early diagnosis and treatment are important to optimize subsequent management and surgical interventions to improve outcomes. Therefore, the search for surrogate markers to predict recurrence is crucial.

Osteopenia, in which bone mineral density (BMD) becomes lower than normal but not as severely low as in osteoporosis, has recently received attention for its association with hepatobiliary cancer [6,7,8]. Dual-energy X-ray absorptiometry (DXA) is the gold standard for evaluating BMD, but computed tomography (CT) scan-based attenuation values are widely used for the preoperative staging of hepatocellular carcinoma patients and are increasingly used to characterize BMD [9]. Sharma et al. recently found that low BMD, a surrogate marker for bone loss, is independently associated with early deconditioning markers that precede sarcopenia [10]. Reports associated with several types of malignancies showed that osteopenia is associated with decreased survival [6,11,12,13]. Bone loss with age was reported to be more common in females as a consequence of post-menopausal findings. It is unclear whether osteopenia can apply to both genders.

Pathological findings are reliable markers for assessing overall survival (OS) and recurrence-free survival (RFS). Regarding histological features, microvascular infiltration and tumor-infiltrating lymphocytes were associated with the risk of recurrence [14]. However, there have been no reports comparing bone loss with pathological findings. The purpose of this study was to assess the prognostic value of osteopenia affecting OS and RFS and to elucidate the association with pathological findings.

## 2. Materials and Methods

### 2.1. Patients

A total of 188 consecutive patients at Jichi Medical University, Japan who underwent liver resection between January 2011 and March 2021 for the treatment of hepatocellular carcinoma were included in this study. The protocol for this study was approved by the ethics committee of Jichi Medical University (approval A22-046) and conforms to the provisions of the Declaration of Helsinki. Written informed consent from any patient for data collection in a prospectively collected database is usually required. However, the necessity for written informed consent for the present study was waived by the Institutional Review Board of Jichi Medical University due to its retrospective design, consistent with national and local guidelines, since clinical/laboratory measurements and procedures were part of routine care.

The hepatectomy technique was classified according to the Brisbane Nomenclature from the International Hepato-Pancreato-Biliary Association [15]. An anatomical hepatectomy was defined as the resection of the tumor together with the related portal vein branches and the corresponding hepatic territory. All non-anatomical hepatectomies were classified as limited resections.

### 2.2. Diagnosis of Osteopenia

CT scan images taken within 3 months before surgery were used for the assessment of BMD. Non-contrast CT scan images at the level of the 11th thoracic vertebra were used. BMD was measured by calculating the average pixel density within a circle at the center of the vertebral body to measure trabecular bone, as described previously [16]. The cutoff value was calculated using a threshold value of 160 Hounsfield units (HU), referring to dual-energy X-ray absorptiometry measurements, because it showed 90% sensitivity for osteoporosis as a reference standard [9,17].

### 2.3. Definition of Sarcopenia and Myosteatosis

Preoperative CT scan images at the third lumbar spine (L3) level were used to measure the psoas muscle mass index (PMI), which indicates low skeletal muscle mass. PMI was calculated by dividing the cross-sectional area of the psoas major muscle by the square of its height (cm^2^/m^2^) [6]. The cutoff values for PMI for gender differences (6.36 for men and 3.92 for women) were set as previously reported by Hamaguchi et al. [18]. Myosteatosis was measured as the region of interest of the multifidus muscle (HU) divided by the region of interest of subcutaneous fat using a preoperative CT scan [16]. The myosteatosis cutoff value was set as −0.457 (area under the curve = 0.48) using a receiver-operating curve analysis of overall survival.

### 2.4. Determination of Prognostic Nutrition Index

The prognostic nutrition index (PNI) was calculated using 10 × serum albumin value (g/dL) + 0.005 × lymphocytes (/mm^3^), with a PNI less than 40 considered impaired, as described in the literature [19].

### 2.5. Pathological Diagnosis

Tissue was fixed with 10% formalin and embedded in paraffin. Hematoxylin–eosin staining was performed using tissue slices 3 μm thick. The pathological specimens were evaluated by pathologists in our hospital based on the World Health Organization Classification of Tumors of the Digestive System (WHO Classification) [20]. If the tumor nodule consisted of multiple histological grades, the highest grade was adopted.

### 2.6. Statistical Analysis

Continuous variables are presented as means ± standard deviation and categorical variables are presented as numerical values. All categorical data were evaluated with Pearson’s chi-squared test. Normally distributed values were analyzed using Student’s *t*-test. Non-normally distributed values were analyzed using the Mann–Whitney U-test. The cutoff values for serum alpha fetoprotein and protein induced by the absence of vitamin K were 10 and 40, respectively, based on hospital reference values. The overall survival curves were constructed using the Kaplan–Meier method. For survival analysis, log-rank tests were conducted using the Kaplan–Meier method. The hazard ratio (HR) and 95% confidence interval (CI) for OS were calculated using Cox’s proportional hazard model. All statistical analyses were performed using JMP version 16.0 (SAS Institute Inc., Cary, NC, USA). The significance threshold was set at *p* < 0.05.

## 3. Results

### Patient Characteristics

The present study included 145 male and 43 female patients. The mean age of non-osteopenic patients was 65.2 ± 10.8 years, and the mean age of patients with osteopenia was 70.8 ± 7.6 years (*p* = 0.0001) (Table 1).

**Figure 1:**
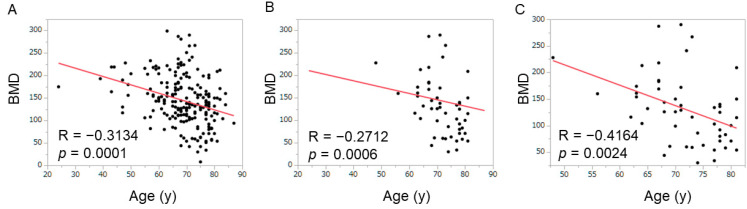
Correlation between bone mineral density and patient age. (**A**) There was a significant correlation between bone mineral density and age in the total cohort (R = −0.3134, *p* = 0.0001). (**B**) In males, there was a significant correlation between bone mineral density and age (R = −0.2712, *p* = 0.0006). (**C**) In females, there was a more significant correlation between bone mineral density and age than in males (R = −0.4164, *p* = 0.0024).

In the regression analysis, age predicted bone mineral density (R = −0.3134, *p* = 0.0001). The predictive power for females (R = −0.4164, *p* = 0.0024) was stronger than for males (R = −0.2712, *p* = 0.0006) (Figure 1).

**Figure 2:**
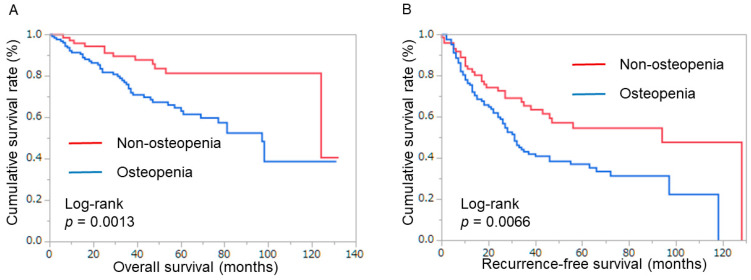
Patient survival in total cohort. (**A**) Overall survival rate after surgery, classified by bone mineral density. (**B**) Recurrence-free survival after surgery, classified by bone mineral density.

The OS of patients with osteopenia was shorter than that of patients with non-osteopenia (5-year OS 81.4% vs. 62.3%, *p* = 0.0013). The RFS of osteopenic patients was shorter than patients without osteopenia (5-year RFS 54.6% vs. 37.7%, *p* = 0.0066) (Figure 2) (Table 2).

**Figure 3:**
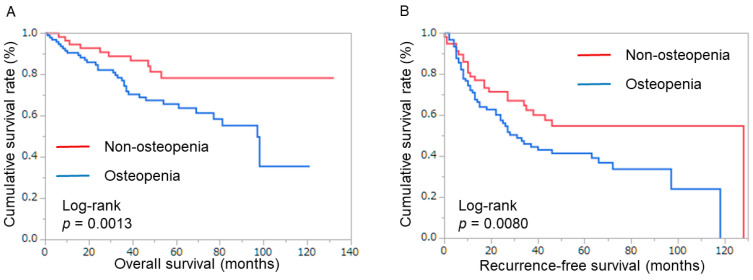
Patient survival in males. (**A**) Overall survival rate after surgery, classified by intramuscular adipose tissue content. (**B**) Recurrence-free survival after surgery, classified by intramuscular adipose tissue content.

Among the male patients, the 5-year OS of non-osteopenic patients increased compared to those with osteopenia (78.4% vs. 62.7%, *p* = 0.0013), as did 5-year RFS (54.8% vs. 42.3%, *p* = 0.008, Figure 3) (Table 2).

**Figure 4:**
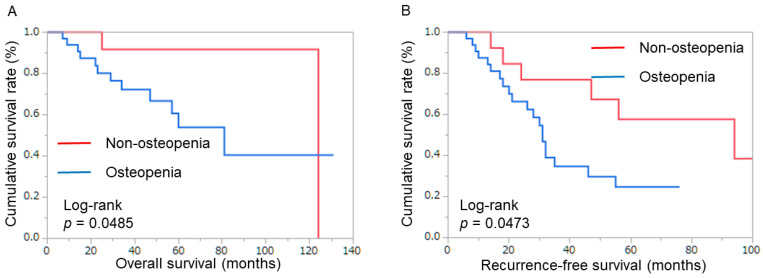
Patient survival in females. (**A**) Overall survival rate after surgery, classified by muscle quantity. (**B**) Recurrence-free survival after surgery, classified by muscle quantity.

The results for non-osteopenia vs. osteopenia were similar in female patients: 5-year OS 91.7% vs. 53.9%, *p* = 0.0485; 5-year RFS 57.7% vs. 24.8%, *p* = 0.0473 (Figure 4) (Table 2).

The univariate analysis shown in Table 3 shows significant hazard ratios (HRs) for the following factors for OS: osteopenia, prothrombin time–international normalized ratio (PT-INR), intraoperative blood loss, γ-glutamyl transpeptidase, aspartate aminotransferase, and operative technique. The multivariate analysis showed that osteopenia was an independent factor significantly associated with OS (HR 2.52, 95%CI 1.32–5.24, *p* = 0.0043).

The univariate analysis with RFS as the outcome (Table 4) showed intraoperative blood loss as the strongest predictor, followed by PT-INR, osteopenia, myosteatosis, and operation time. Osteopenia (HR 1.74, 95%CI 1.12–2.76, *p* = 0.013) was also an independent predictor in the multivariate analysis.

Microvascular portal vein invasion was the sole significant factor in both univariate and multivariate analyses of the pathological factors associated with osteopenia (Table 5).

## 4. Discussion

In the present study, osteopenia was significantly and independently associated with both OS and RFS in both genders. Moreover, osteopenia was associated with microvascular portal vein invasion. This is the first report showing the association between osteopenia and microvascular invasion, which may lead to shorter survival. Several studies have focused on hepatocellular carcinoma with medical treatment other than hepatectomy. In such studies, the comparison before and after treatment can be made due to the presence of the tumor. When studying surgery, the assessment of HCC progression is difficult due to the lack of resected tumors. Therefore, we investigated the pathological findings associated with preoperative bone loss. Microvascular invasion is known to be a prognostic marker leading to poor survival. Moreover, Kang et al. showed that patients with microvascular portal vein invasion exhibited more aggressive tumor characteristics, such as higher tumor marker levels and a higher prevalence of poor histological grade, than patients without microvascular invasion [21].

Sarcopenia has been reported to be a significant risk factor for major organ or vessel invasion, but as yet, osteopenia has not [22]. The risk factors of microvascular invasion in patients with hepatocellular carcinoma are tumor classification, hepatitis B virus and steroid hormone, and tumor capsule [23]. There are relationships between the invaded vasculature and microvascular and the pathological grade of the tumor [23]. Xu et al. showed that epithelial-to-mesenchymal transition (EMT)-related genes, including SNAIL, FoxC1, and vimentin, are upregulated in HCC with microvascular invasion, implying that EMT is associated with the development of microvascular invasion [24]. Receptor activator of nuclear factor kappa B (RANK) is significantly upregulated in human HCC, and receptor activator of nuclear factor kappa B ligand (RANKL) stimulation can lead directly to the migration, invasion, and EMT of HCC cells via NF-kB signaling [25]. RANK exists in muscle and bone and is reported to be associated with sarcopenia and osteopenia. The RANK–RANKL axis may be associated with osteopenia and microvascular invasion.

In the present study, osteopenia was associated with long-term outcomes as well as recurrence-free survival in patients with hepatocellular carcinoma. While minimally invasive resection is considered the treatment of choice for elderly patients with hepatocellular carcinoma, in patients with health problems such as poor nutrition or underlying diseases, the conventional assessment of liver function is not always associated with life expectancy. The cause of this lack of correlation with liver function may be related to a decline in activities of daily living. As osteopenia progresses, it is expected to affect activities of daily living. Mima et al. reported that hepatectomy in patients with hepatocellular carcinoma with reduced activities of daily living can be performed safely, but treatment after recurrence is difficult and has a poor prognosis [26]. Long-term outcomes in patients with hepatocellular carcinoma with reduced activities of daily living may be improved through cancer rehabilitation.

Hepatocellular carcinoma is also prone to recurrence due to its origin in the liver. Radical resection or local treatment is performed in response to these recurrences. It is important to determine whether a patient can tolerate repeat resection and treatment. Osteopenia may affect the ability to tolerate surgery. This is an issue that needs further study.

The association between age and BMD in females is stronger than in males. Various patient-related factors are recognized to influence BMD, including gender, age, race, and menopausal status [8]. In some reports, osteopenia in females was not associated with OS [7,8], possibly because BMD in female patients is so strongly affected by age that its effect on postoperative survival may be diminished [7]. However, in the present study, osteopenia in females was significantly associated with both OS and RFS. One possibility for this discrepancy may be related to differences in the operative techniques used for resection. The present study included anatomic liver resections not included in previous studies [7,8]. Other possibilities for the observed discrepancies may be related to race, distribution of age, and the cutoff value used to define osteopenia.

Intraoperative blood loss is associated with the recurrence of hepatocellular carcinoma and death [27]. Surgical stress, such as hepatic ischemic injury, was reported to promote liver metastases in animal models [28,29]. Remnant liver ischemia was reported to be associated with early recurrence and poor survival after liver resection in patients with hepatocellular carcinoma [4].

In the present study, sarcopenia and myosteatosis were not associated with clinical outcomes in patients with hepatocellular carcinoma. Sarcopenia and myosteatosis are well-known factors associated with frailty. In a previous study, osteopenia was reported to be an independent risk factor for poor prognosis preceding sarcopenia [10]. Previous studies suggests that osteopenia may precede sarcopenia and that this combination may be associated with a poor prognosis [6,10]. Further studies are needed to investigate the impact of osteopenia, sarcopenia, and myosteatosis on outcomes in patients with hepatocellular carcinoma.

The albumin bilirubin (ALBI) score is well known as a prognostic factor in patients with hepatocellular carcinoma; however, it was not significantly associated with prognosis in this study. A previous study was conducted with a large cohort including early- and late-stage patients [30]. The ALBI score may not be suitable for predicting long-term outcomes in patients with hepatocellular carcinoma undergoing hepatectomy.

PT-INR is a standardized index that evaluates coagulation based on the ratio of an individual’s PT to the reference PT. PT-INR and PT are important for assessing the extrinsic coagulation pathway. The elevation of PT-INR or prolonged PT indicates a disturbance in the extrinsic coagulation pathway. The activation of the coagulation system associated with cancer leads to a subsequent deficiency in coagulation factors, resulting in PT prolongation [31]. The decreased capacity of hepatic biosynthesis in patients with cancer may play a role [32]. Previous studies have shown that prolonged PT is associated with a worse prognosis in patients with a variety of malignancies, including lung cancer and hepatocellular carcinoma [33,34]. In the present study, PT-INR was not a significant independent prognostic factor. This discrepancy may be due to differences in patient selection. Most patients included in this study were Child’s Class A, which differs from previous studies [34].

Several limitations of this study should be acknowledged. First, it was conducted retrospectively at a single center. Potential confounders were assessed using descriptive statistics and univariate analysis, and adjustments made whenever possible. In addition, preoperative CT scans and blood tests were routinely performed within 3 months before surgery, limiting the risk of observational bias. The results reported here should be prospectively validated in a multicenter study with a larger study cohort. Second, the cutoff values determined for the diagnosis of osteopenia are based on reports in the literature [6,10], but further studies are needed to verify whether these values are appropriate.

## 5. Conclusions

The loss of bone mass is significantly associated with decreased overall survival in patients undergoing resection of hepatocellular carcinoma, regardless of gender, and is also associated with microvascular portal vein invasion, which may lead to worse survival. Osteopenia may be a surrogate marker of poor prognosis in patients with hepatocellular carcinoma undergoing hepatectomy. Preoperative BMD measurement may be useful for predicting recurrence and be a part of the decision-making process in selecting treatment for patients with hepatocellular carcinoma. The maintenance of activities of daily life with postoperative rehabilitation may enhance resectability for repeat hepatectomy in the case of recurrence.

## Figures and Tables

**Table 1:** Clinicopathological characteristics of patients.

Variables	Non-Osteopenia (*n* = 75)	Osteopenia (*n* = 134)	*p*-Value
Age (years), mean ± SD	65.2 ± 10.8	70.8 ± 7.6	0.0001
Gender, male vs. female	59 vs. 16	100 vs. 34	0.5089
Platelet count, 10^6^/L mean ± SD	1.65 ± 0.62	1.69 ± 0.75	0.6994
PT-INR, mean ± SD	1.09 ± 0.07	1.13 ± 0.23	0.1224
Total bilirubin, mean ± SD	0.85 ± 0.32	0.87 ± 0.39	0.6793
AST, IU/L mean ± SD	44.1 ± 57.1	43.1 ± 27.3	0.8553
ALT, IU/L mean ± SD	43.6 ± 3.9	38.7 ± 2.9	0.3143
PNI, mean ± SD	49.6 ± 5.9	48.2 ± 6.1	0.1175
γ-Glutamyl transpeptidase, IU/L mean ± SD	71.5 ± 76.8	79.6 ± 91.7	0.5208
AFP, IU/L mean ± SD	3171 ± 13,303	3831 ± 22,303	0.8151
PIVKA II, IU/L mean ± SD	1182 ± 4021	1261 ± 3830	0.8898
ALBI score, mean ± SD	−2.82 ± 0.41	−2.72± 0.39	0.1024
LHL15, mean ± SD	0.93 ± 0.004	0.92 ± 0.003	0.4301
Operation method, limited vs. anatomic resection	48 vs. 27	96 vs. 42	0.6485
Intraoperative blood loss, mL, mean ± SD	954 ± 1095	885 ± 1024	0.6485
Operation time, min mean ± SD	314 ± 116	305 ± 111	0.5925

SD, standard deviation; PT-INR, prothrombin time–international normalized ratio; AST, aspartate aminotransferase; ALT, alanine aminotransferase; PNI, prognostic nutrition index; AFP, alpha fetoprotein; PIVKA, protein induced by vitamin K absence; ALBI, albumin bilirubin; LHL15, liver to heart-plus-liver radioactivity at 15 min.

**Table 2:** Overall and recurrence-free survival following surgery for hepatocellular carcinoma with or without osteopenia according to gender.

Variables		5-Year OS	*p*-Value	5-Year RFS	*p*-Value
Total	Osteopenia	62.3%	0.0013	37.7%	0.0066
	Non-osteopenia	81.4%		54.6%	
Male	Osteopenia	62.7%	0.0013	42.3%	0.0080
	Non-osteopenia	78.4%		54.8%	
Female	Osteopenia	53.9%	0.0485	24.8%	0.0473
	Non-osteopenia	91.7%		57.7%	

**Table 3:** Univariate and multivariate analyses of risk factors for overall survival following surgery for hepatocellular carcinoma.

	Univariate Analysis			Multivariate Analysis		
Variables	HR	*p*-Value	95%CI	HR	*p*-Value	95%CI
Age, >70 years	1.29	0.3429	0.76–2.15			
Gender, male vs. female	1.07	0.8268	0.60–2.03			
Platelet count, >10^6^/L	0.98	0.9495	0.48–2.26			
PT-INR, >1.2	2.44	0.0121	1.23–4.46	2.04	0.0569	0.98–3.99
Total bilirubin, >1.5 mg/dL	1.34	0.6365	0.33–3.66			
AST, >37 IU/L	1.67	0.0489	1.00–2.78	1.47	0.1772	0.84–2.59
ALT, >44 IU/L	1.09	0.7485	0.63–1.85			
PNI, >40	0.65	0.1164	0.38–1.12			
γ-Glutamyl transpeptidase, >88 IU/L	1.85	0.0265	1.08–3.12	1.24	0.4766	0.68–2.18
AFP, >10 IU/L	1.55	0.0945	0.93–2.59			
PIVKA II, >40 IU/L	1.19	0.5120	0.71–2.03			
ALBI score, >−2.60	1.24	0.4252	0.72–2.10			
LHL15, >0.91	1.14	0.6552	0.65–2.09			
Operation method, limited resection	0.51	0.0109	0.31–0.86	1.47	0.2260	0.79–2.74
Intraoperative blood loss, >1000 mL	2.15	0.0041	1.28–3.58	1.57	0.1513	0.85–2.91
Operation time, >300 min	1.44	0.1608	0.87–2.44			
Sarcopenia, yes	0.92	0.9059	0.22–3.78			
Myosteatosis, yes	1.62	0.1840	0.80–3,29			
Osteopenia, yes	2.73	0.0008	1.49–5.40	2.52	0.0043	1.32–5.24

PT-INR, prothrombin time–international normalized ratio; AST, aspartate aminotransferase; ALT, alanine aminotransferase; PNI, prognostic nutrition index; AFP, alpha fetoprotein; PIVKA, protein induced by vitamin K absence; ALBI, albumin bilirubin; LHL15, liver to heart-plus-liver radioactivity at 15 min.

**Table 4:** Univariate and multivariate analyses of risk factors for recurrence-free survival following surgery for hepatocellular carcinoma.

	Univariate Analysis			Multivariate Analysis		
Variables	HR	*p*-Value	95%CI	HR	*p*-Value	95%CI
Age, > 70 years	1.27	0.2319	0.86–1.88			
Gender, male vs. female	1.00	0.9833	0.65–1.58			
Platelet count, >10^6^/L	0.75	0.3208	0.45–1.35			
PT-INR, >1.2	1.81	0.0441	1.02–3.02	1.50	0.1530	0.86–2.63
Total bilirubin, >1.5 mg/dL	1.99	0.0905	0.89–3.87			
AST, >37 IU/L	1.05	0.8211	0.70–1.54			
ALT, >44 IU/L	1.10	0.6439	0.74–1.69			
PNI, >40	0.68	0.0681	0.45–1.03			
γ-Glutamyl transpeptidase, >88 IU/L	1.47	0.0816	0.95–2.21			
AFP, >10 IU/L	1.30	0.1935	0.87–1.92			
PIVKA II, >40 IU/L	1.03	0.8897	0.69–1.52			
ALBI score, >−2.60	1.31	0.1881	0.87–1.94			
LHL15, >0.91	0.70	0.0878	0.47–1.06			
Operation method, limited resection	0.88	0.5511	0.74–1.70			
Intraoperative blood loss, >1000 mL	1.99	0.0011	1.32–2.96	1.57	0.0651	0.97–2.53
Operation time, >300 min	1.74	0.0056	1.18–2.62	1.35	0.2195	0.84–2.16
Sarcopenia, yes	0.49	0.3182	0.12–1.99			
Myosteatosis, yes	1.74	0.0473	1.00–3.02	1.52	0.1383	0.87–2.66
Osteopenia, yes	1.79	0.0059	1.18–2.79	1.68	0.0232	1.07–2.62

PT-INR, prothrombin time–international normalized ratio; AST, aspartate aminotransferase; ALT, alanine aminotransferase; PNI, prognostic nutrition index; AFP, alpha fetoprotein; PIVKA, protein induced by vitamin K absence; ALBI, albumin bilirubin; LHL15, liver to heart-plus-liver radioactivity at 15 min.

**Table 5:** Univariate and multivariate analyses of pathological factors associated with osteopenia.

	Univariate Analysis			Multivariate Analysis		
Variables	Odds	*p*-Value	95%CI	Odds	*p*-Value	95%CI
Etiology						
HBV	1.98	0.0853	0.90–4.32	1.82	0.1359	0.82–4.01
HCV	0.87	0.6439	0.49–1.54			
NBNC	1.35	0.3123	0.75–2.43			
Number of tumors						
Solitary vs. multiple	1.08	0.8535	0.49–2.34			
Tumor size, >3 cm	0.78	0.3916	0.43–1.38			
Microvascular invasion						
Microvascular hepatic vein invasion	1.52	0.1636	0.84–2.76			
Microvascular portal vein invasion	2.23	0.0386	1.08–4.59	2.12	0.0415	1.03–4.39
Differentiation						
Poor	0.88	0.7024	0.47–1.66			

HBV, hepatitis B virus; HCV, hepatitis C virus; NBNC, non-B non-C hepatitis virus.

## Data Availability

Our database contains highly sensitive data that may provide insight into clinical and personal information about our patients that leads to their identification. Therefore, according to organizational restrictions and regulations, these data cannot be made publicly available. However, the datasets used and/or analyzed during the current study are available from the corresponding author on reasonable request.
